# Gaming Technology to Support Leisure Activities Among Older Adults

**DOI:** 10.1093/geroni/igaa057.1837

**Published:** 2020-12-16

**Authors:** Walter Boot

**Affiliations:** Florida State University, Tallahassee, Florida, United States

## Abstract

There has been a great deal of research on technology to support older adults in their performance of Activities and Instrumental Activities of Daily Living. There has been substantially less research, however, on exploring technology solutions that support hobbies and leisure. This is unfortunate, as quality of life and well-being are determined by more than just one’s ability to manage everyday tasks. An overview will be presented of research the Center for Research and Education on Aging and Technology Enhancement (CREATE) has conducted over two decades with the goals of understanding and supporting older adults’ performance of technology-based leisure activities. Many of these studies have involved videogaming, where there exists a substantial age-related digital divide. CREATE has evaluated older adults’ attitudes and game experiences through survey and research studies and has even recorded longitudinal gameplay. How these findings can be applied to support technology-based leisure activities will be expanded upon. Part of a symposium sponsored by Technology and Aging Interest Group.

